# Consumers’ perspectives on their involvement in recognizing and responding to patient deterioration—Developing a model for consumer reporting

**DOI:** 10.1111/hex.12858

**Published:** 2018-12-26

**Authors:** Lindy King, Guy Peacock, Mikaila Crotty, Robyn Clark

**Affiliations:** ^1^ College of Nursing & Health Sciences Flinders University Adelaide South Australia Australia; ^2^ Division of Medicine, Cardiac & Critical Care Southern Adelaide Local Health Network Bedford Park South Australia Australia

**Keywords:** consumer education, consumer reporting of patient deterioration, family members, patient safety consumer escalation of care, patients

## Abstract

**Background:**

Adverse events occur in health care. Detection and reporting of deterioration therefore have a critical role to play. Patient and family member (consumer) involvement in patient safety has gained powerful support amongst global policymakers. Few studies, with none taking a rigorous qualitative approach, have drawn upon consumers’ experiences to establish their preferences in consumer reporting of patient deterioration programmes.

**Objective:**

To explore consumers’ experiences of previous reporting of patient deterioration; their preferred educational strategies on this role and recommended pathways in a consumer reporting of patient deterioration model.

**Design, setting and participants:**

An interpretive, qualitative research design was utilized. Nine focus group interviews were undertaken across Adelaide, capital city of South Australia. Interviews were audio‐taped, transcribed and analysed thematically. Twenty‐six adults described, then reflected, on previous experiences of reporting patient deterioration.

**Results:**

Overarching themes incorporated consumers’ experiences and patient/family education. Three themes emerged in relation to consumers’ experiences: feelings, thoughts and actions. Five themes arose on educating consumers: content, timing, format, information providers and information recipients. The consumers’ deep reflections on their past reporting experiences led to the development of a new model for consumer reporting of patient deterioration.

**Conclusions:**

Consumers’ views on ways to improve consumer reporting of patient deterioration processes emerged. These improvements include structured educational programmes for staff advocating open health‐care professional/consumer communication, educational materials developed and tested with English‐speaking and culturally and linguistically diverse consumers and a model with three consumer reporting pathways.

## 1 INTRODUCTION

The need for greater involvement of patients and family members (consumers) in health care related to the field of patient safety has gained powerful recognition amongst global policymakers.^1^, ^2^

Adverse events occur in health care. At the very least, adverse events have led to patients enduring preventable complications, unanticipated transfers to Intensive Care Units, longer hospital stays and diminished capacity for independent living.^3^, ^4^ At their worst, adverse events have been estimated to lead to many preventable hospital‐related deaths globally. Recent evidence has indicated over 200 000 deaths per year relate to medical error in the United States while under‐recognized in other developed countries.^5^, ^6^ Drill‐down analysis of unsafe care incidents resulting in reported deaths in public hospitals in England also pointed to areas of apparent system failure with mismanagement of deterioration, failure of prevention and deficient checking and oversight figuring strongly (72%).^4^

Detection and reporting of deterioration (unexpected decline in physiological condition)^7^ therefore has a critical role to play as early signs of deterioration can often be detected through effective monitoring.^3^, ^7^ The importance of early detection and response to deterioration has been reflected in the emergence of medical emergency teams.^8^ Rapid response systems (RRS) have established processes by which these teams can be activated by health professionals providing swift and intensive medical intervention for deteriorating patients. Call criteria are used to identify patients with conditions that are deteriorating, through abnormal observations and vital signs.^3^, ^9^ Yet evidence of undetected/unreported deterioration has been identified, strongly argued to be related to the impact of socio‐cultural and hegemonic factors on health professionals.^10^, ^11^, ^12^, ^13^ Such findings have led to greater focus on patient safety amongst national health‐care organizations’ responsible for setting policy on health‐care standards and a growing awareness of the potential assistance to be gained by partnering with consumers to detect patient deterioration.^14^, ^15^

A review of evaluative studies undertaken after implementation of a range of consumer reporting of patient deterioration programmes (CRPDP) has indicated consumers can potentially detect and report patient deterioration to RRS.^16^ However, small numbers of consumer reports relating to significant patient deterioration have been reported.^17^, ^18^, ^19^, ^20^ Low levels of consumer knowledge, confidence and/or fear to report in case of upsetting staff may relate to low consumer reporting of patient deterioration.^7^, ^20^ There was little evidence of consumer involvement in planning and designing of these CRPDP.^16^ Greater consumer participation has been sought with the aim to enhance the programmes and increase subsequent consumer involvement in early detection of patient deterioration.^7^, ^21^, ^22^

In the past, health professionals have taken the lead role in development of CRPDP often responding to family demand following tragic, preventable consumer deaths, for example, Josie King.^23^ While input from external consumer organizations into these programmes have been reported,^7^, ^24^ detailed collaborations have been rare with one major exception.^25^ Contrasting with the push toward consumer involvement in CRPDP, studies of patients’ and visitors’ efforts to “speak up” on broad patient safety concerns have indicated fear in doing so.^26,27^ When considering this issue, no studies were found that drew on consumers’ experiences of reporting deterioration to establish their preferences in components of CRPDP.^16^, ^28^

## 2 OBJECTIVE

To explore consumers’ experiences of previous reporting of patient deterioration; their preferred educational strategies on this role and recommended pathways in a consumer reporting of patient deterioration model.

## 3 METHODS

An interpretive, qualitative research design that incorporated focus group methodology^29^ and a consumer‐driven approach was utilized. The study occurred from 2014 to 2017 with interview data from the focus groups audio‐taped, transcribed and analysed by the research team using a manual thematic analysis framework.^30^ Study approval was granted by the Institutional Research Ethics Board. Informed, written consent was obtained from all participants prior to involvement in the study. Reporting of this study has followed the criteria for qualitative research recommended by Tong et al^31^

A purposive sample of participants was sought through community‐based consumer organizations. These organizations shared short articles with members, seeking volunteers for the study. Adults who had been patients or family members of past patients admitted to Australian acute hospitals were sought. Potential volunteers were required to have had experiences of reporting deterioration within 5 years of the commencement of the study. Individuals who had been previous in‐patients for obstetric or mental health reasons were not included as their needs were seen as specialized.

Nine focus groups were undertaken by health‐care researchers experienced in qualitative research methods. Each group was led by one of the three facilitators: a Masters‐prepared hospital‐based quality improvement manager and two University‐based PhD‐prepared senior academics with clinical expertise in cardiovascular and acute surgical care. The 60‐ to 90‐minute interviews were held in hospital or University meeting rooms across Northern, Southern and Central Adelaide settings and via a teleconference for those unable to physically attend. Only research participants and research team members were present at each focus group. Three of the focus groups included both patients and family members, one involved patients only, and five groups were made up of family members only.

The focus group interview approach was based on related literature and guided by Hofmann et al^32^ theories related to cognitive behavioural interaction. Individual's thoughts, feelings and behaviours were asserted to be in a constant state of interaction, with each element influencing the other.^33^ Thus, how an individual interpreted a situation would determine how they experienced that situation on an emotional and cognitive level, then ultimately responded to that situation. The team therefore asked participants to describe their thoughts and feelings then actions taken during detection of patient deterioration.

The focus group interview topic guide was designed to elicit a comprehensive understanding of the patient or family member's experience (Table 1). The focus group interview questions and process were piloted successfully with a volunteer group of eight consumers who provided helpful feedback assisting to finalize the questions. These consumers did not participate in the main study. Examples of the interview questions included “Could you think back to one significant occasion during a hospitalization when you or a loved one suddenly became sicker” and “Who should be given the information about getting help for someone who is becoming physically sicker?” The questions were asked by facilitators with additional questions added to encourage further details (where appropriate). Each participant could also refer to the interview questions on paper and displayed electronically on a computer monitor during the interview. No repeat interviews occurred with the participants.

**Table 1:** Interview topic guide

Consumers’ brief summary of experiences of recognizing and responding to an episode of patient deterioration: What they felt What they thought What they did in response to their concern What would have improved their experience
Consumers’ views on changes needed within hospital systems to make it easy for patients or family members to: Identify and report patients who are physically deteriorating Ensure timely assistance for patients from health professionals
Consumers’ reflections on potentially receiving information on how to recognize and respond to deterioration in future: Who should provide this information When participants should be given this information What multimedia formats should provide the information Who should receive this information

The focus group interviews were audio‐recorded, transcribed by a professional transcriber and de‐identified as “FG” 1‐9 with pages numbered. The basis of each focus group's discussion was the participants’ recollections and subsequent responses to each other's comments. Interview transcripts were therefore not sent to participants. Previous in‐patients were identified within the transcripts as “P” (n = 9) and family members of in‐patients as “FM” (n = 17).

Six phases of exploratory thematic analysis^30^ were utilized in the manual analysis of the interview data. Phase one involved the research team familiarizing themselves with each interview as two to three of the four members were present at each focus group. The decision was made to combine the patients and family members’ data in the analysis for two reasons. The first reason was based on the decision to bring patient and family members into the same focus groups (due to participants’ availability). The second reason was the patient, and family member data were found to be strikingly similar across all of the focus groups. Initial codes were then generated related to features of the data that were of specific interest to the researchers (examples included in Table 2). These segments of data emerged as common responses (phase two). The codes were grouped into potential themes by a single coder and then confirmed by the team. Data extracts were identified by patient or family member acronyms (P or FM), focus group (FG) and by page number of transcript (phase three).

**Table 2:** Extract of data with codes

Data extract	Codes
I was angry, feeling helpless. People telling me I didn't know what I was talking about [concerning mother], so frustrated (FM,FG3,p3)	Feelings of anger and helplessness during report Frustrated by perceived dismissal of knowledge
[son's] fever wasn't very high at all and then all of a sudden it just spiked (FM,FG4,p.3)	Mother's close attention to change of signs in child
They're way understaffed, rushed off their feet. I found it hard, I just felt like a burden, I didn't want to buzz (P,FG,p34)	Perceived busyness of understaffed health professionals Patient not wanting to be a burden to busy staff
Nurse said ‘do you want a MET call for your [mother].’ Luckily I understood what she meant and said yes (FM,FG1,p9)	Report led to involvement in escalation of care decision Drew on own knowledge of health‐care systems to escalate

Themes were reviewed in relation to phrases used by individuals and compared across the interview data set. Preliminary thematic mapping then commenced (phase four). Refinement of the themes occurred through re‐reading of participants’ experiences and reflections. Data saturation occurred when no new emergent themes or subthemes were noted following analysis of all of the interviews (phase five). Two overarching themes were clear, related to the participants’ experiences when reporting deterioration and reflections on patient/family education on their potential role in reporting of patient deterioration. Themes related to consumers’ experiences incorporated feelings, thoughts and actions. Themes associated with patient/family education included content, timing, format, information providers and information recipients. Finally, the participants’ preferences on how to improve the process of reporting deterioration by patient or family member generated a new model for consumer reporting of patient deterioration (phase six).

Guba and Lincoln^33^ advocate trustworthiness of qualitative research in terms of credibility, transferability, dependability and confirmability. Accurate representation of the participants’ views was critical with member checking identified as one way of assuring credibility. To this end, verbal checks were undertaken with consumers during each interview. Each consumer confirmed the interviewer's understanding and volunteered additional explanation whenever queries arose. No further feedback was therefore required from the participants following the interviews. Transferability, the degree of resonance between the participants’ experiences and perspectives and that of others in similar situations has been made easier to gauge through detailed description. Dependability, transparency of the research process, has emerged through: recording and transcribing of the interviews; provision of a clear, replicable description of the data analysis and use of interview quotes to illustrate emergent themes. Demonstration of credibility, transferability and dependability has facilitated confirmability, establishing that the findings of this study have emerged from the consumers’ views.

## 4 RESULTS

Twenty‐six (26) participants (19 women and seven men) volunteered. The participants included 17 family members and nine patients who were residents of metropolitan Adelaide or nearby regions of South Australia. Ages ranged from 27 to 86 years, providing perspectives across generations. A profile of the patients’ reason for hospitalization (when known) and nature of deterioration episodes has been summarized in Table 3.

**Table 3:** Reason/s for hospitalization and nature of deterioration episode

P/FM, FG	Reasons for hospitalization (relationship to participant)	Nature of deterioration episode
FM, FG1	Meningococcal disease (daughter)	Severe headache/vomiting/40° temperature/tachycardia/low blood pressure
FM, FG1	Pneumonia (husband)	Pain in side/Cognitive impairment/Physical collapse
FM, FG1	Pneumonia/Acute Pulmonary Oedema/Heart failure (mother)	Breathlessness
P, FG2	Staphylococcus aureus/golden staph/Diabetes (self)	Gangrenous foot
P, FG2	Hysterectomy/Postop bleeding (self)	Vaginal blood loss
P, FG2	Lap band surgery/Pulmonary embolism (self)	Severe chest pain/breathlessness/feeling unwell
FM, FG2	Staphylococcus aureus/golden staph/Diabetes (relative)	Gangrenous foot
FM, FG3	Brain tumour/craniotomy (mother)	Physical collapse during rehabilitation session
FM, FG3	Lung cancer (mother)	Increased breathlessness/tachycardia
FM, FG3	Stroke (father)	Worsening of symptoms (left side facial drooping, no strength in left side)
FM, FG4	Retrocaecal appendicitis (son)	Spike in fever
FM, FG4	Traumatic lung injury (brother)	Difficulty in breathing
FM, FG4	Hip operation (daughter)	Unrelieved postoperative pain
P, FG5	Investigation/lobectomy for lung cancer (self)	Detection of ongoing respiratory symptoms at home
P, FG5	Perianal abscess (self)	Increased pain/increased bleeding
P, FG5	Eye surgery (self)	Unspecified complications of surgery/Postoperative low blood pressure
P, FG6	Epidural abscess and septicaemia/staph infection (self)	Inability to walk/extreme fatigue/excruciating pain/high fever
P, FG6	Pleurisy and pneumonia (self)	Sudden, sharp stabbing chest pains
FM, FG6	Epidural abscess and septicaemia/staph infection (wife)	Inability to walk/extreme fatigue/excruciating pain/high fever
FM, FG7	Fall/hip fracture/postop respiratory complication (father)	Difficulty breathing/change in appearance
FM, FG7	Fractured hip/followed by stroke (father)	Loss of movement in legs/loss of consciousness
FM, FG7	Fall/Physical collapse (father)	Suicide attempt by starvation
FM, FG8	Myocardial infarction (husband)	Increased chest pain
FM, FG8	Ruptured appendix/Peritonitis/bladder laceration (wife)	Severe abdominal pain/vomiting/inability to digest food/weight loss
FM, FG9	Investigation/diagnosis of Lung cancer/Pneumonectomy (daughter)	Watching for deterioration in early postoperative period
P, FG9	Appendicitis/Ruptured appendix/peritonitis (self)	Severe abdominal pain/vomiting/inability to digest food/weight loss

During the interviews, participants described occasions involving either themselves or a family member's deterioration requiring rapid medical intervention. The emergent themes focused on Consumers’ experiences when reporting deterioration and Patient/Family education—information on recognition and reporting of patient deterioration. A model for consumer reporting of patient deterioration was also developed subsequent to the initial thematic analysis. The two themes are presented first in the results section followed by a description of the new Model.

### 4.1 Consumers’ experiences when reporting deterioration

**Figure 1:**
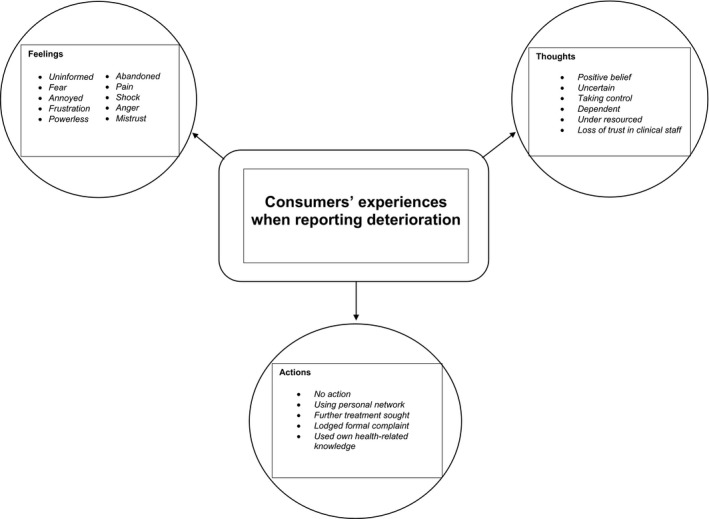
Consumers’ experiences when reporting deterioration

This theme has been constructed from the consumers' feelings, thoughts and actions which have been described below, accompanied by evocative responses (please also refer to Figure 1). A more extensive summary of the participants’ comments on each of these themes and subthemes can be found elsewhere (Table S1).

### 4.2 Feelings

On detection and reporting of deterioration, each participant's feelings were related to the health‐care professional's perceived lack of response to their concern. Experiences ranged from feeling uninformed to fear, annoyed, frustration, powerless, abandoned, in pain to shock, anger and mistrust in the health‐care staff. As one patient commented: “My primary feeling was fear. I knew something was going on and felt they weren't validating that concern” (P,FG 2,p6).

### 4.3 Thoughts

When considering the health professional's response to their report of deterioration, some participants, like the patient below, held positive thoughts about the clinician's assessment and provision of the necessary care.

I thought I would be alright, they looked like they knew what they were doing; it was the best place to be (P,FG2,p7).

Others were uncertain about how to engage with clinicians to gain fast action, describing their thinking as dependent and powerless, perceiving the health system as under resourced with weak care coordination.

They're way understaffed, rushed off their feet. I found it hard, I just felt like a burden, I didn't want to buzz (P,FG,p34).

Contrastingly, some decided to try to take control of the situation. These participants drew on their medical knowledge to successfully escalate concern about the patient. Confident participants sought advice from the senior nurse and, as a result, gained collaborative involvement in decision making on the need for a call to the rapid response team. As one commented:

[the] Nurse kept going back to the senior who realised I was making a bit of a fuss and said ‘do you want a MET call for a relative’. Luckily I understood what she meant and said yes (FM,FG1,p9).

Some participants described an eventual loss of trust when they thought clinicians were not addressing their concern. As one participant said:

I felt very torn between believing that these are professionals, they must know what they're doing, we just have to trust them, and seeing what I was seeing in front of me, and seeing that incongruity between people telling us that it's okay and that it's not looking okay, it's actually looking worse than I've ever seen it (FG8, FM, p. 9).

### 4.4 Actions

The participants’ actions after reporting deterioration were diverse. Some described taking no further action beyond reporting their concern. Others drew on their own knowledge and skills to seek action through the system. Several participants described tapping into their own personal network for guidance on gaining further treatment.

If I hadn't known people…the rapid assessment team came because my friend [spoke up]. I don't know what would have happened. Time was a big thing with [child's] illness (FM,FG1,p12).

A number of participants eventually lodged formal complaints in regard to their efforts to report patient deterioration. As one patient stated:

I wrote a letter of complaint and got a reply. They did say that it was wrong, they're now using my case as a blind study. [but] The letter is really saying, ‘there, there dear, it's alright, nobody else will get treated the way you did’ (P,FG6,p14).

### 4.5 Patient/Family education—information on recognition and reporting of patient deterioration

**Figure 2:**
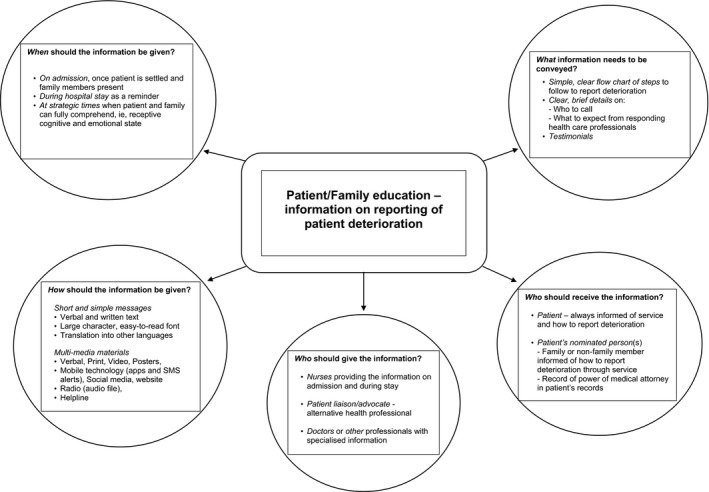
Patient/Family education—information on reporting of patient deterioration

The participants then offered their perceptions on the most effective ways to inform consumers on their potential role of reporting deterioration (Figure 2). This theme has been constructed from the consumers’ preferences for information delivery on their potential reporting role, described below, and accompanied by participants’ quotations. A more extensive summary of quotes made by consumers on each of the subthemes within the theme can be found elsewhere (Table S2). The five themes were: “*what information should be conveyed”* (Content), “*when the information should be given”* (Timing), “*how the information should be given?”* (Format), “*who should provide the information”* (Information providers) and “*who should receive the information”* (Information recipients).

### 4.6 Content

The participants felt the position of the consumer to report should be made clear in the content of the information provided. A simple flow chart of steps to follow to obtain further assistance, clear details on who to call when raising concerns and what to expect from clinicians responding to their concerns were sought. As one patient described, content should be “Simple [in order] to make it easy to read and absorb” (P,FG4,p5).

### 4.7 Timing

The consumers wanted to receive the information on admission, regularly during their hospital stay, and at strategic times when patients and family members could fully comprehend, that is, where they were in a receptive cognitive and emotional state. Typically, as one patient stated: “On admission when family members were waiting, would be an ideal time” (P,FG6,p14).

### 4.8 Format

A multimedia approach was recommended, incorporating communication methods that would reach across cultures and age groups. Consumers with low health literacy were seen as particularly vulnerable. Participants wanted information presented positively, leading them to feel safe. Preferred multimedia modes included: *Verbal explanation,* considered paramount, participants described the struggle to process printed information when feeling distressed, necessitating verbal communication. *Print* format, as brief information to support verbal explanation, was favoured. *Video* materials were very popular through a range of devices and platforms. Videos were recommended for adults and children in the form of real life‐experiences or realistic role plays where consumers successfully reported and received assistance for patient deterioration. *Posters* were deemed related to printed information but thought to function differently. Consumers sought their strategic placement in patients’ rooms, wards, waiting rooms and toilets. Mobile technology through smart phones and tablet devices were seen as an excellent medium to enhance the communication process through various approaches (e.g, apps, SMS alerts or websites). *Other forms of communication* included alternate verbal and written formats for accurate information. Overall, participants suggested that hospitals needed to be “sensitive to knowing when people are cognitively and emotionally ready to receive information” (FM,FG8,p22).

### 4.9 Information providers

Nursing and medical staff were the preferred professionals to provide information on the consumer's potential role in reporting deterioration. However, further education of health professionals to enhance effective communication with consumers was recommended. Key communication skills sought in health professionals were their ability to: listen and acknowledge family member's knowledge of the person; respect for the consumer's ability to provide vital contextual information; give clear explanations and feedback on the patient's condition (without assumption of consumer's level of health literacy); clarify family members’ potential roles; choose the right time to communicate and ensure a senior clinician assess the patient.

*Nurses* were seen as the preferred professional to provide information to consumers; nurses currently on the shift were seen as responsible for taking on that initiative.

At times, the terms “*liaison”* and “*nurse”* were used together with a specialist nurse liaison recommended. The consumers sought access to a patient liaison or advocate with advanced assessment skills as someone they could turn to who also had in‐depth knowledge of the health‐care system.

A liaison to turn to [would be helpful] as it's a big decision to make when you're feeling disempowered (FM,FG4,p11).

*Medical and other health‐care professionals* were also seen as potentially effective providers of information; doctors for medical‐related questions. Participants described the need for other forms of assistance, particularly in situations that were less urgent. For example, social workers to handle family resource needs and chaplains for spiritual support. As one family member commented: “Social workers are better equipped to handle family members and find resources” (FM,FG1,p3).

### 4.10 Information recipients

Participants felt that the *patient* should always be informed and able to nominate *a family member or friend* to receive information about their potential role in reporting patient deterioration. For example, one family member viewed this formal recognition in the health system as important so the nominee “can recognise when they're getting sicker and press the button” (FM,FG3,p8). Participants also recommended respect for power of medical attorney, allowing that person to access information and advocate while the patient was incapacitated.

### 4.11 Model for consumer reporting of patient deterioration

**Figure 3:**
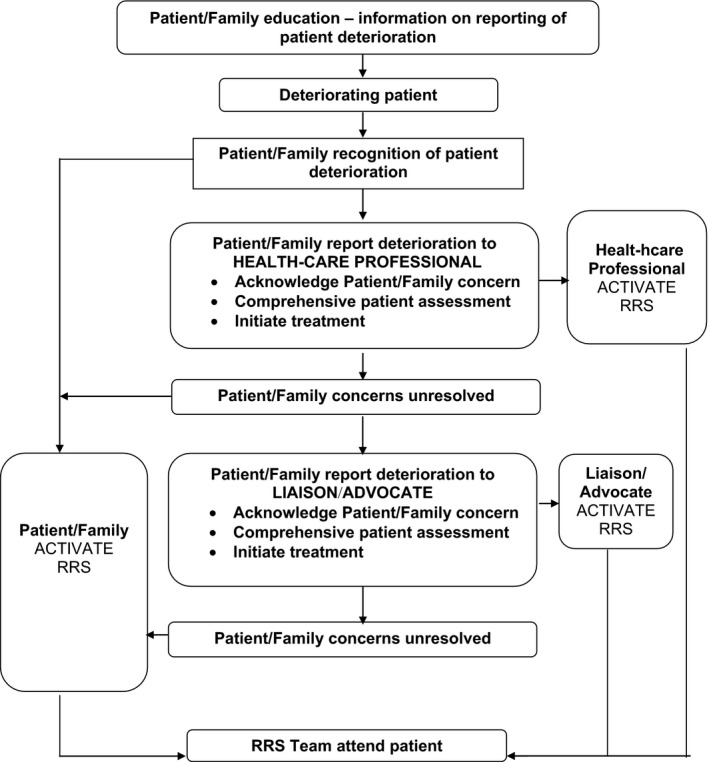
Model for consumer reporting of patient deterioration. RRS, rapid response system

The consumers’ perspectives then guided development of an innovative model for consumer reporting of patient deterioration (see Figure 3). This model became known by the catchphrase, “*You're Worried, We're Listening”* that emerged from the consumers’ comments. This catchphrase exemplified their desire for respectful, two‐way communication when reporting their concerns about patient deterioration to health professionals. The proposed model (Figure 3) has three reporting pathways for consumers following recognition of patient deterioration.

#### 4.11.1 Direct report to a RRS team of a patient found in an unexpected state of severe deterioration

Consumers sought the opportunity for direct activation of the RRS team by ringing a specific phone number of the hospital switchboard. Circumstances for direct RRS activation were recommended when the patient was in an unexpected life‐threatening situation.

#### 4.11.2 Direct report to the health‐care professional/s involved in the care of the patient

Direct reporting of the consumer's concerns to a health professional involved in the patient care was the most frequent pathway recommended in CRPDP.^7^, ^21^, ^24^, ^25^

#### 4.11.3 Direct report to a patient liaison or advocate who could assess the patient

The consumers sought access to a patient liaison or advocate with professional knowledge to assess the patient and call the RRS team if required. Particularly when the consumer's concern was not alleviated by the initial patient review and response from the health‐care professional(s). A health‐care professional in a liaison role from within the hospital but outside the ward/unit setting was recommended. Critical care‐based nurse responders with advanced life support skills and a designated role to assess patients at the bedside and activate the RRS team as needed have been utilized in outreach models.^7^ However, smaller or regional health‐care organizations may need to access other health professionals through remote services.

To be satisfied with the overall process of CRPDP, family members wanted their reporting to elicit rapid skilled treatment to address reversible clinical patient deterioration. Importantly, the consumers were well aware that patients may die during critical illness or be faced with a transition into end‐of‐life care.

## 5 DISCUSSION

The aim of this study was to investigate consumers’ past hospital experiences to inform development of a consumer‐directed model for reporting patient deterioration. This study explored consumers’ experiences of reporting previous patient deterioration; consumers’ views on consumer‐targeted educational information on reporting patient deterioration; and the preferred processes for consumers to report and escalate care for a deteriorating patient.

In regard to communicating with health professionals, our findings were consistent with previous research indicating the reticence of consumers to “speak up” on patient safety concerns in health‐care.^26^, ^27^, ^34^ The confidence of consumers in our study to speak about their concerns was carefully weighed up against the: potential harm of doing so; importance of their concerns opposed to other patients’ needs; perceived staff workloads and knowing how to “navigate” the health‐care system. Consumers in other studies have also indicated greater likelihood to “volunteer their concerns if staff actively seek their views.”^27^

Effective consumer/health‐care professional communication has become known as crucial to the achievement of patient safety.^1^, ^35^, ^36^ A structured educational programme advocating open health‐care professional/consumer communication, prior to introduction of the consumer reporting model, was recommended by the consumers and strongly supported elsewhere.^7^, ^21^, ^24^, ^25^ Many consumers felt fear of meeting resistance in staff to listen and respond to their concerns.^26^ Further education of health‐care staff to remind them of the importance of effective listening and responding when communicating with consumers has been recommended.^25^ It was believed that consumer education to report patient deterioration could not hope to succeed unless health professionals were prepared to listen and respond effectively.

Our proposed consumer reporting model starts with the education of the consumers (illustrated in Figure 3 and described in the findings). The consumers in this study underlined the critical importance of receiving educational materials to build their confidence and knowledge to report. Historically, as well as currently, educational materials for consumers on reporting have been/are developed by groups of health professionals.^37^ In contrast, consumers in this study sought materials developed and tested with consumers themselves on the basis that they may achieve a better understanding of the messages and effective response amongst visitors to the health‐care organization. These consumers’ views echo the strong push toward widespread consumer collaboration in development of materials and CRPDP.^16^, ^25^, ^38^ Similarly, consumers’ recommended educational materials be developed and evaluated with culturally and linguistically diverse consumers; few studies have focused on this aspect of reporting programmes.^21^, ^39^

The proposed model has three potential reporting pathways for consumers following recognition of patient deterioration. The first pathway, “Direct report to a RRS team of a patient found in an unexpected state of severe deterioration,” has been found in current use in several health‐care organizations who provide consumers with access to phone numbers that can lead to activation of RRS teams.^21^, ^25^ Will all consumers be prepared to participate in reporting? Residual reluctance in some consumers due to socio‐cultural norms appears likely.^27^, ^40^ Longtin et al^40^ list of factors that could influence consumer participation in decisions related to patient safety deserves serious consideration when educating consumers on potential reporting.

The second pathway, “Direct report to the health‐care professional/s involved in the care of the patient”, was the most frequent pathway recommended in CRPDP.^7^, ^21^, ^24^, ^25^ We found the third pathway, “Direct report to a patient liaison or advocate who could assess the patient” emerged as a particularly interesting point. The consumers sought access to a patient liaison or advocate with a very advanced level of professional knowledge to assess the patient and call the RRS team if required. This pathway was sought when the consumer's concern was not alleviated by the initial patient assessment/response by the health‐care professional. A health‐care professional in a critical care‐based liaison role from within the hospital but outside the ward/unit setting was recommended. Smaller, regional and remote health‐care organizations may require access to advanced health‐care professionals via electronic/technological services. Critical care‐based nurse responders with advanced life support skills and a designated role to assess patients at the bedside and activate the RRS team as needed have been utilized in outreach models.^7^ Liaisons were found in the form of administrative managers who could assess patients and report to the appropriate department.^20^ However, no further studies of consumer reporting models were identified that offered consumer access to a critical care RN as an advocate, separate from the full RRS team, for assessment of patient deterioration on the wards.

Overall, the aim of the study was achieved, that is, the development of a consumer‐informed model for reporting of patient deterioration. An in‐depth understanding of consumers’ needs in relation to educational materials on the reporting process was also gained. The need for educational programmes for staff advocating open health‐care professional/consumer communication was also very apparent. All of our findings point toward consumers’ growing demand for a partnership driven approach to health‐care delivery.

### 5.1 Limitations of the study

Consumers chose to participate in this study based on their own previous experiences of patient deterioration in hospital as a patient or family member. Their experiences provided a strong basis for reflection on difficulties met in reporting their own or a relative's deterioration and receiving rapid and effective medical response. It was noteworthy that none of the reported experiences involving patient deterioration preceded the person's death.

While a small number (26) in quantitative terms, the participants ranged in age, gender and residential location providing potentially diverse views through rich qualitative data. Transferability of these findings rests on the meaningfulness of the consumers’ perspectives to others in similar settings.

### 5.2 Recommendations for policy, clinical practice, education and further research

The proposed model has the potential to be used within departments of health policy to guide consumer reporting of patient deterioration programmes required by national safety and quality health‐care service standards in Australia and other developed countries.^14^ Following implementation of the proposed model in practice, evaluation research will be needed. Part of the evaluative process will involve the measurement of consumer knowledge and confidence to report deterioration and development of educational materials for consumers on their potential role. We recommend that consumers should be involved in the development and testing of educational materials to accompany new programmes. The educational needs of consumers with limited English language skills will also require further research. Research into health‐care professionals’ views on consumer reporting would also be beneficial to inform staff education during implementation of the new programme. Openness to a partnership between health‐care professionals and consumers in the use of the model would signify a much needed move away from the current professional‐centric approach.

## 6 CONCLUSIONS

The outcomes of this study have shown that based on experience consumers have strong opinions on how reporting of the deteriorating patient can be improved. These improvements include structured educational programmes for staff advocating open health‐care professional/consumer communication, educational materials developed and tested with English‐speaking and culturally and linguistically diverse consumers and a model with three consumer reporting pathways.

## CONFLICTS OF INTEREST

None of the authors have conflicts of interest.
