# Supplementary material for: Consumers’ perspectives on their involvement in recognizing and responding to patient deterioration—Developing a model for consumer reporting
Source: Health Expect. 2018 Dec 26;22(3):385–95. doi: 10.1111/hex.12858 (PMC6543137; doi:10.1111/hex.12858)
Supplement: Supplementary file 1 [file HEX-22-385-s001.docx]

| **S1-Online Supplementary Information: Consumers’ experiences when reporting deterioration** | |
| --- | --- |
| **Themes** | **Participants’ quotes** (P/FM, FG/page) |
| **Feelings** |  |
| *Uninformed* | They never told me until towards the end, ‘oh yeah and it was meningococcal.’ I felt a bit uninformed (FM,FG1,p44) |
| *Fear* | My primary feeling was fear. I knew something was going on and felt they weren't validating that concern (P,FG 2,p6) |
| *Annoyed* | Everybody blamed my foot on diabetes but it was to do with an infection. That's what annoyed me (P,FG2,p12) |
| *Frustration* | I was angry, feeling helpless. People telling me I didn't know what I was talking about, so frustrated (FM,FG3,p3) |
| *Powerless* | It's a big decision especially when you're feeling disempowered, Do I ask for more help? (FM,FG4,p18) |
| *Abandoned* | I felt that you're just stuck in a room, no one pokes their head in except to give medication (P,FG5,p11) |
| *Pain* | She was getting smaller, thinner, paler, having pain, receiving a lot of painkillers - we were concerned (FM,FG8,p4) |
| *Shock* | I woke up and couldn't breathe. I finally coughed and choked and spluttered and lay there in shock (P,FG9,p25) |
| *Anger* | Powerless, frustrated and angry, I became demanding-they weren't listening, taking over but not helping (P,FG6,p9) |
| *Mistrust* | I didn't trust that that was correct. I was shattered to see my dad not being cared for as I asked (FM,FG3,p5) |
| **Thoughts** |  |
| *Positive belief* | I thought I would actually be alright, they looked like they knew what they were doing; it was the best place to be (P,FG2,p7) |
| *Uncertain* | I kept questioning myself, if they say everything's okay, even though I feel really bad they must know (P,FG2,p7) |
| *Taking control* | Nurse kept going back to the senior who realised that I was making a bit of a fuss and said ‘do you want a MET call for a relative’. Luckily I understood what she meant and said yes (FM,FG1,p9) |
| *Dependent* | I didn't feel I could push them otherwise they would throw me out and say ‘no you're distressed and not rational’ (FM,FG3,p6) |
| *Under resourced* | They're way understaffed, rushed off their feet. I found it hard, I just felt like a burden, I didn't want to buzz (P,FG,p34)  I was thinking that they should have some senior staff or proper staff monitoring the patients (FM,FG1p9) |
| *Loss of trust in clinical staff* | I was devastated and I didn't trust that that was correct but I had to have a certain amount of faith in the people caring for him. I have to trust in that because I don't know any better myself (FG 3,FM,p6)  I don't trust them, they're in a very powerful position - making decisions on your behalf that might not be the best (FG 6,P,p8) |
| **Actions** |  |
| *No action* | There was nothing I could do. I couldn't create an operating theatre. I couldn't take him to another hospital. I couldn't do anything and that was the hard part (FM,FG4,p7) |
| *Used own health-related knowledge* | She was in with pneumonia and I found her in heart failure. The nurse, I don’t think she had the clinical abilities to pick that mum was crook. I did with my medical background and I’d also been dealing with mum’s issues for a long time (FM,FG1,p8) |
| *Using personal network* | If I hadn’t known people; the rapid assessment team came because my friend [spoke up]. I don’t know what would have happened. Time was a big thing with [child’s] illness (FM,FG1,p12) |
| *Further treatment sought* | They said, there's nothing wrong with her. I took her to our GP who put her on antibiotics and said, we'll have her seen by an infection specialist’. Then told ‘if she hadn't have gone there and got this treatment, she would have been dead’ [FG6_P3_p11] |
| *Lodged formal complaint* | I wrote a letter of complaint to [hospital] and got a reply. They did say that it was wrong, they're now using my case as a blind study. The letter is really saying, ‘there, there dear, it's alright, nobody else will get treated the way you did’ (P,FG6,p14) |
