# Supplementary material for: Consumers’ perspectives on their involvement in recognizing and responding to patient deterioration—Developing a model for consumer reporting
Source: Health Expect. 2018 Dec 26;22(3):385–95. doi: 10.1111/hex.12858 (PMC6543137; doi:10.1111/hex.12858)
Supplement: Supplementary file 2 [file HEX-22-385-s002.docx]

| **S2-supplementary information: Patient/Family education – information on reporting of patient deterioration** | |
| --- | --- |
| **Themes** | **Participants’ quotes** (P/FM, FG/page) |
| **Content** |  |
|  | Not too much information (FM,FG3,p7) |
|  | Simple to make it easy to read and absorb (P,FG4,p5) |
|  | Provide different translations for those with limited English reading skills (FM,FG3,p8) |
|  | Simple signs to look out for and then ring for your nurse or go to someone (P,FG2,p6) |
|  | Rights, people don't know that private surgeons go to public hospitals. You can actually get your GP to refer (FM,FG4,p52) |
|  | That it is all right to say there's something wrong and feel okay about that (FM,FG3,p23) |
| **Timing** |  |
|  | On admission when family members were waiting, would be an ideal time (P,FG6,p14) |
|  | A reminder post-admission (P,FG6,p14) |
|  | Being sensitive to knowing when people are cognitively and emotionally ready to receive information (FM,FG8,p22) |
| **Format** |  |
|  | We all relate to different mediums (FM,FG7,p20) |
|  | Be ready to deal with people from various cultures and ages (FM,FG7,p21) |
|  | Consumers with low health literacy are particularly vulnerable (FM,FG4,p11) |
| *Verbal* | Should be between nurse, patient and family-address all your questions and they can respond (FM,FG8,p23) |
|  | She had trouble understanding so they needed to try different ways to explain (FM,FG3,p9) |
|  | You're worried about your child or family member, so if someone actually spoke, you can take that in (FM,FG4,p11) |
| *Print* | Although intellectually I knew, I really did need to see [it] in writing (P,FG5,p15) |
|  | In addition to someone who would come and sit down and talk to you about it (P,FG2,p4) |
| *Video* | On a loop in a GP office (FM,FG8,p22) and Waiting rooms in the hospital (P,FG2,p6) |
|  | At the patient’s bedside (P,FG2,p6) |
|  | Tailored for adults and children as real life experiences from former patients or realistic role plays (P,FG2,p5) |
|  | Public mediums such as YouTube (P,FG6,p16) or live television in the form of an infomercial (FM,FG3,p7) |
| *Poster* | Strategic placement in patient rooms, wards, waiting rooms, on the back of toilet doors (FM,FG3,p9) |
|  | Act as a guide but also take away the fear that you're imposing, that you're interrupting busy work (FM,FG8,p23) |
|  | You can get information on a wall that says ‘we’ll help you, if you think the situation is urgent, tell someone’ (FM,FG1,p3) |
|  | Visual cues to remind people (FM,FG4,p12) |
|  | Just a poster on the wall that says something like, ‘do you feel worse than you did an hour ago’? (P,FG2,p5) |
|  | A hospital could have an app - how this hospital communicates with you (FM,FG7,p19) |
| *Mobile technology* | If there's a hospital app and you get that information, then you know what to do (FM,FG8,p23) |
|  | Social media (P,FG2,p6) |
|  | Recommended websites [because] you don't know what is a credible source of information (FM,FG3,p8) |
| *Other formats* | Helplines (FM,FG7,p20) |
|  | Radio (P, FG6, p16) |
| **Information providers** |  |
| *Nurses* | Registered or senior nurse (P,FG2,p4) |
|  | Nurse liaison recommended to bring it all together (FM,FG1,p3) and to go in and have the discussion together (FM,FG3,p9) |
|  | Nurse because they understand the situation and what's going on (FM,FG4,p11) |
| *Doctors* | To address medical-related questions (FM,FG4,p10) |
| *Liaison* | Patient liaison/advocate with knowledge of the hierarchies and who to talk to (FM,FG3,p7) |
|  | A liaison to turn to as it's a big decision to make when you're feeling disempowered (FM,FG4,p11) |
| *Others* | Social workers are better equipped to handle family members and find resources (FM,FG1,p3) |
|  | Chaplains for spiritual support (FM,FG4,p10) |
| **Information recipients** |  |
| *Patient* | So they can recognise when they're getting sicker and press the button (FM,FG3,p8) |
| *Nominated person* | Are you happy for [name] to be given this information, should your situation deteriorate, how to report it (FM,FG3,p7) |
|  | When a person is admitted, ‘I give authority for my contact person to be kept informed about my condition’ (FM,FG7,p19) |
